# Dr. Ure

**Published:** 1857-04

**Authors:** 


Dr. Ure.—We have to record the death of this eminent man, who died February 2d, 1857. Dr. Andrew Ure was born at Glasgow, May 10, 1778; he studied at the University of his native town, and afterwards at that of Edinburgh.

He graduated in medicine at Glasgow, 1801, having previously obtained the degree of master of arts; in 1804 was appointed professor of chemistry at the Andersonian University of Glasgow; in 1822 was elected a Fellow of the Royal Society. Dr. Ure was one of the original Fellows of the Geological Society. We subjoin a list of his most prominent literary labors.

Dictionary of Chemistry, first published 1821, of which many editions have since appeared.

A Translation of Berthollet on Dyeing. 2 vols., 8 vo. 1824.

A New System of Geology. 1829.

The Philosophy of Manufactures. 1835.

The Cotton Manufactures of Great Britain compared with that of other countries. 1836.

Dictionary of Arts, Manufactures, and Mines, 1837. To which was added in 1839, a Supplement; and in 1853, was greatly enlarged.

